# Urinary Peptides As a Novel Source of T Cell Allergen Epitopes

**DOI:** 10.3389/fimmu.2018.00886

**Published:** 2018-04-26

**Authors:** Ricardo da Silva Antunes, John Pham, Curtis McMurtrey, William H. Hildebrand, Elizabeth Phillips, Simon Mallal, John Sidney, Paula Busse, Bjoern Peters, Véronique Schulten, Alessandro Sette

**Affiliations:** ^1^La Jolla Institute for Allergy and Immunology, La Jolla, CA, United States; ^2^Department of Microbiology and Immunology, University of Oklahoma, Oklahoma City, OK, United States; ^3^Institute for Immunology and Infectious Diseases, Murdoch University, Perth, WA, Australia; ^4^Department of Medicine, Vanderbilt University School of Medicine, Nashville, TN, United States; ^5^Division of Clinical Immunology, Icahn School of Medicine at Mount Sinai, New York, NY, United States; ^6^University of California San Diego School of Medicine, La Jolla, CA, United States

**Keywords:** urine, peptides, T cell, allergen, mouse, epitopes

## Abstract

Mouse allergy in both laboratory workers and in inner-city children is associated with allergic rhinitis and asthma, posing a serious public health concern. Urine is a major source of mouse allergens, as mice spray urine onto their surroundings, where the proteins dry up and become airborne on dust particles. Here, we tested whether oligopeptides that are abundant in mouse urine may contribute to mouse allergic T cell response. Over 1,300 distinct oligopeptides were detected by mass spectrometry analysis of the low molecular weight filtrate fraction of mouse urine (LoMo). Posttranslationally modified peptides were common, accounting for almost half of total peptides. A pool consisting of 225 unique oligopeptides of 13 residues or more in size identified within was tested for its capacity to elicit T cell reactivity in mouse allergic donors. Following 14-day *in vitro* stimulation of PBMCs, we detected responses in about 95% of donors tested, directed against 116 distinct peptides, predominantly associated with Th2 cytokines (IL-5). Peptides from non-urine related proteins such as epidermal growth factor, collagen, and Beta-globin accounted for the highest response (15.9, 9.1, and 8.1% of the total response, respectively). Peptides derived from major urinary proteins (MUPs), kidney androgen-regulated protein (KAP), and uromodulin were the main T cell targets from kidney or urine related sources. Further *ex vivo* analysis of enrichment of 4-1BB expressing cells demonstrated that LoMo pool-specific T cell reactivity can be detected directly *ex vivo* in mouse allergic but not in non-allergic donors. Further cytometric analysis of responding cells revealed a bone fide memory T cell phenotype and confirmed their Th2 polarization. Overall, these data suggest that mouse urine-derived oligopeptides are a novel target for mouse allergy-associated T cell responses, which may contribute to immunopathological mechanisms in mouse allergy.

## Introduction

The kidneys filter unwanted substances from the blood and produce urine to excrete them. There are three main steps of urine formation: glomerular filtration, reabsorption, and secretion. These processes ensure that only waste and excess water are removed from the body. Nearly all proteins and oligopeptides are reabsorbed, preventing them from disappearing from the body through the urine. The molecular weight cutoff for glomerular filtration is thought to be 30–50 kDa (1).

In humans, the excretion of more than 0.5 mg of protein daily (proteinuria) is considered abnormal and of potential pathological significance. For example, determination of peptide excretion rates by filtration through a 10 kDa molecular-weight-cutoff membrane showed that albumin and other protein-derived peptides are excreted at high concentrations (up to 4 g/day) in the urine of patients with diabetes (2).

The urine protein content of mice, in contrast to most other mammals, is high (3), and proteinuria as a normal condition was recognized as early as 1933 (4–6). The high protein content of mouse urine has consequences in terms of the induction of allergic reactions in humans exposed to mice. Mice spray urine onto their surroundings, and as the proteins dry up, they become airborne on dust particles (7). Mouse urine contains specific proteins, termed major urinary proteins (MUP), which are involved in mouse-to-mouse communication and territorial marking (7–9). MUP, because of their structure, relatively small size, and biological function as olfactory proteins, are volatile. It is thought that their inhalation is a contributor to their high allergenic potential (10, 11).

Mouse urine is indeed the most prominent source of murine allergens, which have been described over two decades ago (12, 13). In addition to Mus m1, several new mouse proteins have recently been shown to be human T cell targets in mouse allergy (14–16). High concentrations of airborne rodent allergens in laboratories and inner-city apartments (17–19) have been associated with allergic rhinitis, allergic conjunctivitis, and high incidence of asthma (20–23), and thus constitute a serious public health concern.

Because allergens in general are defined on the basis of IgE reactivity, it is not surprising that all mouse allergens are proteins with a molecular weight of 10 kDa or higher (24). However, mouse urine is also an abundant source of relatively small molecular weight peptides, either derived from the breakdown of proteins or actual excretion. The mouse urine peptidome has been characterized by mass spectroscopy (9, 25).

Alongside the well-studied IgE-mediated allergic reaction (24), T cell reactivity and recognition of allergen T cell epitopes also plays an important role in the sensitization and potentiation of allergic reactions (16, 26–29). Small peptides in the urine are potentially volatile and could be adsorbed through the nose and mucosal surfaces. While their small size would make them unlikely to contribute to IgE responses, peptides naturally bound to HLA class II molecules and recognized by human helper T cells are about 15 residues in size. Thus, recognition of peptides derived from mouse urine by specific T cells could represent a novel contributor to overall adaptive immune responses associated with inhalant allergens.

## Materials and Methods

### Study Population and PBMC Isolation

A cohort of 29 mouse-sensitized patients, as defined by mouse-specific IgE titers of >0.35 kU_A_/l, was studied. A cohort of 10 non-allergic and non-exposed subjects, defined by the lack of mouse-specific IgE titers (<0.35 kU_A_/l) and no known exposure to mouse allergens was used as a negative control. Individual subjects were recruited from San Diego, CA, USA, and New York City, NY, USA following Institutional Review Board approval (IRB protocols: VD-112-0217, GCO 13-0691). All subjects enrolled in this study provided written consent. Demographic and clinical information is summarized in Tables S1A–C in Supplementary Material. IgE titers were determined from plasma by ImmunoCAP (ThermoFisher, Uppsala, Sweden). PBMCs were isolated from whole blood by density gradient centrifugation according to manufacturers’ instructions (Ficoll-Hypaque, Amersham Biosciences, Uppsala, Sweden).

### Urine and Epithelia Mouse Allergen Extracts

Mouse epithelial extract (EPTH) was purchased from Greer (Lenoir, NC). Mouse urine (mixed gender pooled, unfiltered) was purchased from CliniScinces (Nanterre, France). Low molecular flow-through components (<3 kDa) were isolated by filtration centrifugation using Amicon Ultracel tubes (Merck Millipore, Darmstadt, Germany) of mouse urine and further analyzed by 2D LCMS to identify specific peptide sequences, after de-salting gel chromatography. The high molecular weight fraction (>3 kDa) was washed six times with PBS, each time followed by repeated centrifugation in Amicon Ultracel tubes with a cutoff of 3 kDa. Protein/peptide content was determined by Pierce BCA Protein Assay Kit (ThermoFisher). The resulting urine high molecular weight (PUR) and low molecular weight (FLT) extracts were lyophilized and subsequently resuspended in PBS.

### Mass Spectrometry Analysis

Lyophilized mouse urine flow-through was resuspended in 10% acetic acid and loaded onto a reverse-phase column (pore size, 90 Å; 2 mm i.d. by 150 mm long; Jupiter 4 µm Proteo; Phenomonex) with a Michrom Paradigm MG4 high-pressure liquid chromatograph (HPLC) and eluted at pH 10 with an organic gradient of 2–10% acetonitrile for 2 min followed by 10–60% acetonitrile for 60 min in water with 40 mM ammonium bicarbonate. Fractions were collected every minute resulting in 49 peptide-containing fractions. Each of these fractions was dried and resuspended in 10% acetic acid and placed into an Eksigent NanoLC 400 U-HPLC auto sampler system (Sciex). Approximately 20% of each fraction was injected onto a nano-LC trap column (C18 trap column, 350 µm i.d. by 0.5 mm long with 3-µm particles and 120-Å pores, ChromXP) and washed for 5 min in solvent A (98% water, 2% actetonitrile, 0.1% formic acid).

The peptides eluted from the trap column were put in line with a separation column (C18 trap column, 75 µm i.d. by 15 cm long with 3-µm particles and 120-Å pores, ChromXP). The mobile phase solvents were solvent A (98% water, 2% actetonitrile, 0.1% formic acid) and solvent B (5% water, 95% actetonitrile, 0.1% formic acid). The elution consisted of two linear gradients of solvent A and B: 10–40% solvent A for 70 min then 40–80% solvent B for 10 min. Eluting peptides were ionized with a nanospray III ion source and analyzed with a 5600 Triple-TOF mass spectrometer (Sciex). Survey and fragment spectra of eluting ions were collected with data-dependent acquisition (DDA) in positive ion polarity.

Peptide sequences were derived from the DDA spectra using PEAKS 7.0 software (Bioinformatic Solutions). Fragment spectra were searched against the Uniprot murine (*Mus musculus*) proteome with a precursor mass error tolerance of 50 ppm and a fragment mass error of 0.05 Da. Variable posttranslational modifications consisted of: cysteinylation (C), hydroxylation (D, K, N, P, R, Y), dihydroxy (F, K, P, R), oxidation (M, W, H), acetylation (N-term, R), deamidation (N, Q), pyroglutamate from glutamic acid, and sodium adducts (D, E, c-term). A false discovery rate (FDR) was estimated for assigned sequences using a target-decoy fusion approach provided by PEAKS 7.0. All sequences were assigned at a 1% FDR.

### Peptide Synthesis and LoMo Megapool Generation

225 oligopeptides identified within the low molecular weight filtrate fraction of mouse urine were synthesized by A and A (San Diego, CA, USA) as crude material on a small (1-mg) scale. Individual peptides were resuspended in DMSO at a final concentration of 40 mg/ml. The peptides were pooled, lyophilized, and the resulting megapool (LoMo) was resuspended to a final concentration of 1 mg/ml.

### HLA Typing and Inferred Restriction

Donors were HLA typed either at the La Jolla Institute or by an ASHI-accredited laboratory at Murdoch University (Western Australia). HLA typing was performed for Class I (HLA A; B; C) and Class II (DQA1; DQB1, DRB1 3,4,5; DPB1) using locus-specific PCR amplification on genomic DNA. Primers used for amplification employed patient-specific barcoded primers. Amplified products were quantitated and pooled by subject and up to 48 subjects were pooled. An unindexed (454 8-lane runs) or indexed (8 indexed MiSeq runs) library was then quantitated using Kappa universal QPCR library quantification kits. Sequencing was performed using either a Roche 454 FLX^+^ sequencer with titanium chemistry or an Illumina MiSeq using a 2 × 300 paired-end chemistry. Reads were quality-filtered and passed through a proprietary allele calling algorithm and analysis pipeline using the latest IMGT HLA allele database as a reference. Potential HLA-epitope restriction odds ratios and relative frequencies were calculated using the RATE program (30).

### Stimulation and Expansion of T Cells With LoMo Megapool

For *in vitro* expansion of LoMo-specific T cells, PBMCs of mouse-sensitized individuals were stimulated with LoMo megapool at a final concentration of 1 µg/ml. Stimulation concentrations to induce optimal T cell responses were previously determined by titration (data not shown). Cells were cultured in RPMI 1640 supplemented with 5% human AB serum in 24 well plates (BD Bioscience, San Diego, CA, USA) at a density of 2 × 10^6^/ml and incubated at 37°C. IL-2 was added every 3 days after initial stimulation. Cells were harvested on day 14 and screened for IFNγ and IL-5-production by ELISPOT.

### Dual ELISPOT Assays

The production of IFNγ and IL-5 from cultured PBMCs in response to antigenic stimulation was assessed by dual ELISPOT assays as described previously (28). Cells (1 × 10^5^ cells/well) were stimulated with either peptide pools (1 µg/ml), individual peptides (10 µg/ml), or PHA (10 µg/ml), or medium containing DMSO corresponding to the DMSO % in the pools/peptides as a control. Spot-forming cells (SFC) were counted by computer assisted image analysis (KS-ELISPOT reader, Zeiss, Munich, Germany). Criteria for positivity were ≥20 SFCs per 10^6^ PBMCs, *p* < 0.05 by Student’s *t*-test or by a Poisson distribution test, and a stimulation index ≥2. Positive pools (≥100 SFC) were deconvoluted to identify the individual epitopes inducing the response.

### IgE Reactivity Against PUR Extract, LoMo Peptides, and Epidermal Growth Factor (EGF) Restriction of Dominant Epitopes

LoMo peptides (10 μg/ml), high molecular weight (PUR) extract (10 μg/ml), and mouse rEGF (ThermoFisher) (2.5 μg/ml) were incubated overnight at 4°C and then blocked with 1% BSA in PBS for 2 h at room temperature. Plasma was allowed to react for 2 h at room temperature with mild agitation. IgE was detected by incubation with HRP-conjugated antihuman IgE monoclonal antibody for 1 h at room temperature (ThermoFisher). The plates were developed by addition of the TMB substrate. The absorbance at 450 nm was measured with a VersaMax microplate reader (Molecular Devices, Sunnyvale, CA, USA). A standard curve was run in parallel with purified antihuman IgE as a capture antibody and titration of a recombinant human IgE.

### Activation-Induced Marker (AIM), ICS, and 4-1BB Enrichment Assays

The AIM assay was previously described (31). This assay detects cells that are activated as a result of antigen-specific stimulation by upregulation of activation-induced surface markers. Here, we employ 4-1BB (CD137) enrichment (CD137 MicroBead Kit, Miltenyi, Germany) followed by staining with a cocktail containing CD4-APC-ef780, CD3-AF700, CD8/CD14/CD19-V500, CD45RA-eF450, CCR7-PerCPCy5.5, 4-1BB-APC, and OX40-PECy7 (see Table S2 in Supplementary Material). Cryopreserved PBMCs were thawed, and 1 × 10^6^ cells/condition were stimulated with LoMo megapool (2 µg/ml) or high molecular weight (PUR) urine extracts (10 µg/ml) in 5% human serum (Gemini Bioproducts) for 24 h. DMSO (0.25%) with medium was used as negative control. For intracellular cytokine staining (ICS), PBMCs were incubated with LoMo megapool or PUR extract for 24 h. After 20 h, BFA [5 μg/ml (BD Bioscience, San Diego, CA, USA)] was added for an additional 4 h. Cells were then washed, stained for extracellular markers for 30 min, washed again, fixed with 4% paraformaldehyde, permeabilized with 0.5% saponin (Sigma), and stained for intracellular IL-4-BV421, IL-5-PE, IL-17-FITC, and IFN-γ-PerCPCy5.5 (Table S2 in Supplementary Material). PMA and ionomycin (1 and 0.1 µg/ml) were used as positive control. Samples were acquired on a BD LSRII Flow Cytometer and analyzed using FlowJo X Software.

## Results

### Identification of Oligopeptides From Mouse Urine Filtrate

In the context of an independent study to characterize conventional murine allergens, we prepared and purified mouse urine extracts. The purification process entailed repeated ultracentrifugation steps through a 3 kDa Centricon membrane. The total protein content of the ultrafiltrate (endproduct after six washes and filtration steps) derived from 60 ml of urine was 162.8 mg by BCA assay. In contrast, the protein content of the seven filtrates flow-through samples yielded a total of 347 mg. These data suggest that a large amount of protein/peptide/amino acid content in the mouse urine comes from low molecular weight material, consistent with previous reports (8, 25, 32). Comparative gel analysis of whole urine to the purified/desalted protein sample did not reveal significant differences both in terms of identity and quantity of the bands suggesting that the lower content of BCA-reactive material in the ultrafiltrate was not due to protein loss during the ultrafiltration process (data not shown).

**Figure 1:**
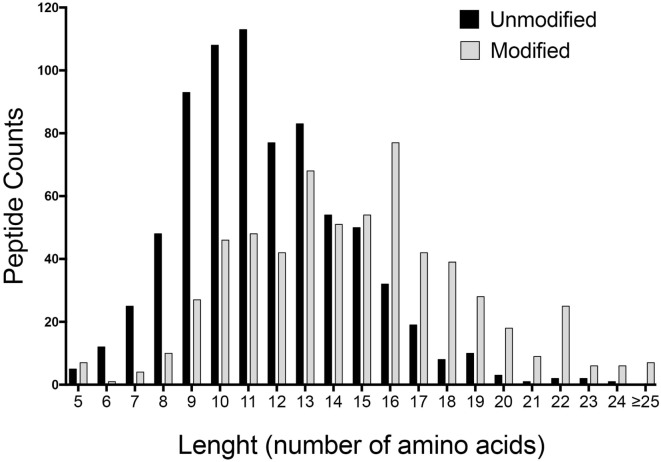
Length distribution of unmodified and modified identified peptides. Liquid chromatography–mass spectrometry of <3 kDa mouse urine fraction detected over 1,300 distinct peptiforms. Graph depicts the summary of the amino acid length distribution of non-modified (746) and modified peptides (615) at a high confidence level.

We therefore hypothesized that the BCA-detected material of less than 3 kDa in size could be accounted for, at least in part, by oligopeptides. Peptides greater than 9 residues in size are recognized by T cells but will be missed by any analyses based on IgE binding. To address this point, we performed 2D LCMS to identify specific peptide sequences, after de-salting the sample by gel chromatography. Spectra were searched against the *M. musculus* database using a set of variable common posttranslational modifications. This analysis detected over 1,300 distinct peptiforms with 746 non-modified and 615 modified peptides at a high confidence level (Table S3 in Supplementary Material). Furthermore, most of the peptiforms (1,129) were >9 amino acids in length, with a median of 15 and 11 amino acids for modified and unmodified peptides, respectively. A summary of the length distribution of all identified peptides is shown in Figure 1.

In all, there were 265 unique non-modified peptides of 13 or more residues in size. Many of these peptides were largely overlapping with other peptides derived from the same source protein. For example, four peptides of different lengths from Mep1a were observed (YSGDNDAILEWPVENR, YSGDNDAILEWPVENRQ, YSGDNDAILEWPVENRQA, and YSGDNDAILEWPVENRQAI). These four peptides were accordingly grouped in a single “cluster” represented by the YSGDNDAILEWPVENRQAI species. After this clustering reduction was completed, there were 164 clusters of one or more peptides representing 164 different locations across 54 source proteins. A total of 21 proteins contributed 10 or more peptiforms, suggesting that the peptides were derived from a relatively narrow set of proteins (Table S4 in Supplementary Material). By far, the most common posttranslational modification observed was hydroxyproline (42%), and most of these peptides originated from collagen (Figure S1 in Supplementary Material). Indeed, we detected over 300 collagen derived peptides associated with mono- or di-hydroxylated prolines, also consistent with previous reports reporting that hydroxylated collagen peptides are relatively abundant in urine (33). Accordingly, we next synthesized the longest peptide from each of the 164 clusters and 61 hydroxyproline peptides. In all, this corresponds to 225 peptides derived from the low molecular weight fraction of mouse urine, hereafter as “LoMo” peptides.

### Urine Low Molecular Weight Fraction (LoMo) Peptides Are Recognized by T Cells From Mouse Allergic Donors

Next, to determine whether LoMo peptides were recognized by human T cells, we assessed their antigenicity in PBMC from a cohort of 19 mouse-sensitized donors (Table S1 in Supplementary Material). A total of 225 peptides from 68 proteins were screened after PBMC expansion with pools of LoMo peptides.

**Figure 2:**
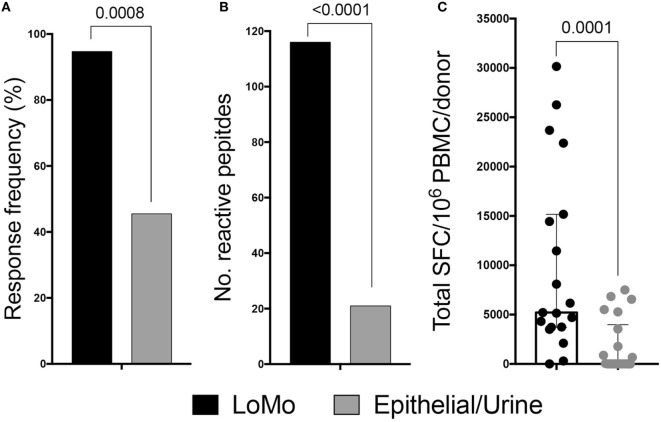
LoMo peptides induce antigen-specific responses for T cells derived from mouse allergic donors. Cytokine production was measured by ELISPOT following *in vitro* restimulation with LoMo peptides or urine/epithelial extract. Donor response frequency **(A)** and breadth of responses **(B)** were compared between antigenic stimulation by exact fisher test. **(C)** Magnitude of responses expressed by spot-forming cells (SFC). Median is shown. Each dot represents combined IL-5 and IFNγ responses of one donor. Statistical analysis was performed by two-tailed Mann–Whitney test.

**Figure 3:**
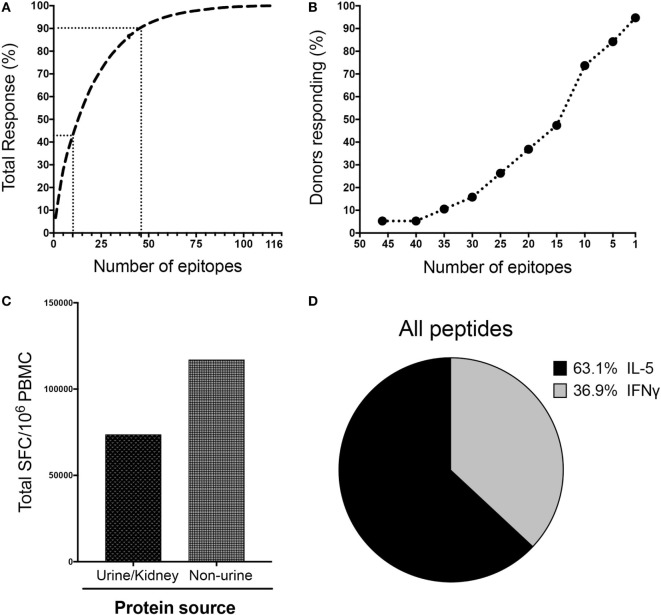
LoMo immunodominance, protein source, and response polarization. **(A)** Epitopes ranked on the basis of magnitude of response. Dotted lines indicate the top 10 (42.6% of total response) and top 46 (90% of total response) epitopes. **(B)** Breadth of response indicated by proportion of donors who respond to the specified number of epitopes. **(C)** Total spot-forming cells (SFC) detected against peptides from urine/kidney or non-urine protein sources. **(D)** Pattern of cytokine production expressed as proportion of IFNγ or IL-5 for all the reactive peptides.

Strong reactivity was detected and directed against a total of 116 peptides (Table S5 in Supplementary Material). The overall response frequency was 94.7% (18/19 donors), and the median total response magnitude (sum of IFNγ and IL-5) was 5,210 SFC/donor (Figure 2). The results were initially depicted as sum of IFNγ and IL-5 to simplify the flow of the manuscript. The relative contribution of IFNγ and IL-5 to total reactivity is detailed in Figure 3D below which addresses polarization of the responses.

As a control, the same 225 peptides were screened after 14-day *in vitro* expansion with either mouse high molecular weight purified urine (PUR) or mouse epithelial (EPTH) extract. In the experiments in Figure 2, PBMCs were expanded for 14 days with LoMo peptides before testing for reactivity against the LoMo peptides. Expansion with epithelial/urine extract before testing against the LoMo peptides was also used as a control; since the LoMo peptides were not contained in the PUR/EPTH extract we expected that LoMo reactive T cells would not be effectively expanded. Thus, we expected lower reactivity, potentially indicative of some degree of cross-reactivity between high molecular weight products and LoMo peptides. Whenever possible, the same donors also tested for reactivity to the LoMo peptides were utilized for these control experiments. In some cases, however, limitations in cell numbers did not allow for testing the same donors, in which cases additional donors with same clinical characteristics were tested, to provide a comparable number of donors tested with LoMo and the high molecular weight extracts (see Table S6 in Supplementary Material for a complete listing of the peptides for which responses were detected following expansion with high molecular weight extracts).

Following PUR/EPTH expansion and assessment of reactivity against LoMo peptides, the overall response frequency was 10/22, which is significantly lower (*p* = 0.0008 by the exact Fisher test) compared with LoMo expansion (Figure 2A). In addition, breadth of recognition (no. of epitopes) was also significantly reduced (21 peptides after PUR/EPTH expansion vs. 116 peptides after LoMo expansion) (*p* < 0.0001 by the exact Fisher test) (Figure 2B), and the median total magnitude of responses was also significantly lower compared with LoMo expansion (*p* = 0.0001 by the Mann–Whitney test) (Figure 2C).

These results demonstrate that LoMo peptides are potent inducers of T cell reactivity in mouse allergic donors and that the observed responses are largely not due to epitope cross-reaction with high molecular weight extract products, but instead to original epitopes identified within the low molecular weight fraction of mouse urine.

### Characterization of LoMo Responses in Terms of Immunodominance, Protein Source, and Response Polarization

We next scrutinized the specificity of LoMo responses in more detail. The top 46 epitopes accounted for 90% and the top 10 epitopes for 42.6% of the total magnitude of the detected responses (Figure 3A). As shown in Figure 3B, 94.7% of donors recognized at least one epitope, and 73.7% of the donors responded to ≥10 epitopes.

LoMo responses, on a per donor basis, tended to be distributed among a relatively high number of recognized peptides (median of 13 epitopes/donor). Indeed, a total of 50 epitopes were recognized in at least 3 out of the 19 donors tested, corresponding to a frequency of recognition of 15.8% (see Table S5 in Supplementary Material). Moreover, 15 epitopes were recognized in 31.6% of the donors (6/19 positives). Interestingly, within the top 46 peptides accounting for 90% of the total response, 5 peptides contained hydroxyproline residues, and 1 particular hydroxyproline peptide elicited the highest magnitude of response (7% of total response).

In terms of protein source, Table S5 in Supplementary Material also lists whether the proteins from which the peptides are derived are urinary/kidney proteins or not. Strikingly, the majority (94/116) of the immunogenic peptides were derived from non-urine related sources representative of 36 different proteins. These peptides accounted for a higher magnitude of responses (61.4%) than urine and kidney related proteins (Figure 3C). Major urinary proteins, KAP, leucine-glycoprotein, uromodulin, collagen, Meprin A, and Egf have already been reported previously in urinepeptidome studies (25, 34, 35). However, for other proteins, like Lacrein or Beta-globin, this is the first time that peptides from these protein sources have been reported to be present in urine, as well as being targets of T cell reactivity in mouse allergic donors. The data shown in Tables 1 and 2 emphasizes that the high amount of non-urine sources contributes to T-cell responses, as also mentioned earlier in Figure 3C. SFC of each individual peptide across all donors was summed as groups of peptides from same protein source. The % total response for a given protein is the relative contribution of that protein in the overall responses for the entire sources of proteins.

**Table 1:** Protein sources of urine or kidney reactive peptides.

Urine or kidney related proteins	Response frequency (%)	Total spot-forming cells	% of Total response
Major urinary protein 13	84.2	42,878	22.5
MCG15829, isoform CRAa (major urinary protein 25) (major urinary protein 3)	42.1	10,825	5.7
Kidney androgen-regulated protein (ARP) (KAP)	52.6	9,437	5.0
Major urinary protein 14 (major urinary protein 17)	31.6	5,183	2.7
Major urinary protein 20 (Darcin) (major urinary protein 24)	15.8	2,985	1.6
Uromodulin (Tamm–Horsfall urinary glycoprotein) (THP)	21.1	2,317	1.2

**Table 2:** Protein sources of non-urine reactive peptides.

Non-urine proteins	Response frequency (%)	Total spot-forming cells	% of Total response
Egf protein	52.6	30,330	15.9
Collagen and collagen precursors	26.3	17,485	9.2
Beta-globin	26.3	15,478	8.1
Meprin A subunit alpha (EC 3.4.24.18) (endopeptidase-2) (MEP-1)	36.8	9,063	4.8
Putative uncharacterized protein	36.8	6,822	3.6
Leucine-rich HEV glycoprotein (leucine-rich alpha-2-glycoprotein) (protein Lrg1)	52.6	6,315	3.3
Cfd protein [complement factor D (Adipsin), isoform CRA_a]	10.5	4,167	2.2
Lacrein (protein Gm1553)	52.6	3,722	2.0
Pancreatic alpha-amylase (PA) (EC 3.2.1.1) (1,4-alpha-d-glucan glucanohydrolase)	36.8	2,708	1.4
Fibrinogen alpha chain (cleaved into: fibrinopeptide A; fibrinogen alpha chain)	15.8	2,653	1.4
Fibronectin	10.5	2,547	1.3
Protein Bpifb9b	21.1	2,493	1.3
Alpha-1-antitrypsin 1-3 (alpha-1 protease inhibitor 3) (Serpin A1c)	26.3	2,370	1.2
Beta-geo	15.8	1,723	0.9
SH3 domain-binding glutamic acid-rich-like protein 3	21.1	1,597	0.8
Insulin-2 (fragment)	15.8	923	0.5
Tripeptidyl peptidase I, isoform CRA_a	15.8	913	0.5
Alpha-2-HS-glycoprotein (countertrypin) (Fetuin-A)	26.3	810	0.4
Igk protein	31.6	775	0.4
Cadherin 1, isoform CRA_a (Cdh1 protein)	26.3	632	0.3
Hemoglobin	10.5	600	0.3
Seminal vesicle secretory protein 4 (seminal vesicle protein 2) (SVS IV)	10.5	595	0.3
Vasodilator-stimulated phosphoprotein (VASP)	10.5	475	0.2
Cathelicidin (fragment)	10.5	370	0.2
Deoxyribonuclease-1 (EC 3.1.21.1) (Deoxyribonuclease I) (DNase I)	5.3	240	0.1
Serine protease inhibitor A3K (Serpin A3K) (contrapsin) (SPI-2)	5.3	210	0.1
Asialoglycoprotein receptor 1 (asialoglycoprotein receptor 1, isoform CRA_b)	10.5	173	0.1
ATP-binding cassette sub-family A member 13	10.5	150	0.1
Lysosomal thioesterase PPT2 (fragment)	15.8	120	0.1
Beta-2-microglobulin	10.5	103	0.1
Espin	10.5	97	0.1
Apolipoprotein E	5.3	83	0.04
Carbonic anhydrase 1 (EC 4.2.1.1) (carbonate dehydratase I) (carbonic anhydrase I) (CA-I)	5.3	57	0.03
Apolipoprotein A-II (Apo-AII) (ApoA-II) (apolipoprotein A2)	5.3	53	0.03
Alpha-1-acid glycoprotein 2 (AGP 2) (orosomucoid-2) (OMD 2)	5.3	50	0.03
Acetylcholinesterase collagenic tail peptide	5.3	27	0.01

Allergic T cell response is known to be predominantly associated with responses of the Th2 subset (36, 37). Here we sought to further characterize the polarization of LoMo-specific T cell responses based on IFNγ/IL-5 production per epitope tested. As expected, T cell reactivity in response to stimulation with the individual peptides was dominated by IL-5 production, which accounted for 63.1% of the total responses (Figure 3D). The sum of IFNγ and IL-5 responses for all the reactive peptides across all donors defines the value accounting for 100% of responses. The relative contribution of either IFNγ or IL-5 production is represented in the pie chart as the fraction of responses accounted by each cytokine. Double positive cells (producing both cytokines) were generally not observed. The *ex vivo* responses results presented below in Figure 6C also support this notion.

### IgE Reactivity Against PUR Extract, LoMo Peptides, and EGF Restriction of Dominant Epitopes

**Figure 4:**
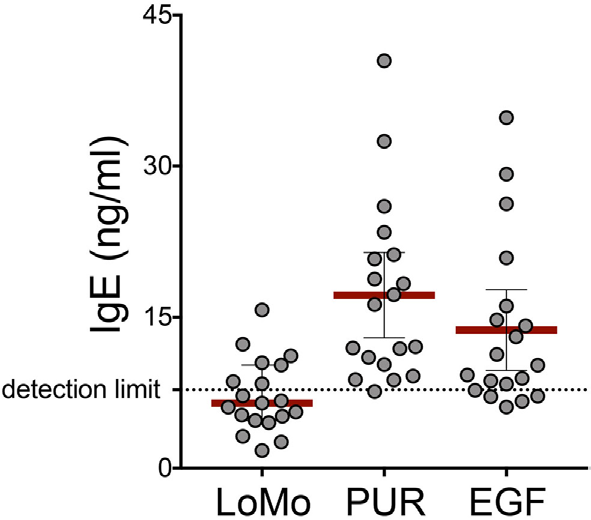
IgE reactivity against PUR extract, LoMo peptides, and epidermal growth factor (EGF). IgE from plasma of mouse allergic donors was measured by ELISA against coated LoMo peptides, urine high molecular extract (PUR) fraction, or rEGF. Concentration values for each condition were extrapolated from a calibration curve generated with recombinant IgE titrations read in the same plate. Values represent the average of duplicates, and each dot represents one donor. Detection limit (7.8 ng/ml) represents the sensitivity of the assay.

Further experiments addressed the IgE reactivity of LoMo peptides, tested as a pool, and reactivity against EGF, the most reactive non-urine protein from which the LoMo was derived. The rationale for these experiments was first to demonstrate that, as expected LoMo peptides were, because of their small size, largely non-IgE reactive. Furthermore, testing for recognition of a representative full-length protein was of interest in terms of the characterization of these novel responses, to examine whether also antibodies against the corresponding novel proteins exist. The results show that as expected, good reactivity was noted against the positive control, the high molecular extract (PUR) fraction of the urine (Figure 4), but the LoMo peptides were largely non-reactive. Interestingly, reactivity was also detected against EGF, suggesting that the LoMo peptides might actually also be linked to the identification of novel IgE reactive proteins.

### Inferred HLA Restriction of Dominant Epitopes

HLA typing was performed as described in the Methods (Table S7 in Supplementary Material). To analyze the potential relation between the HLA class II genotype of subjects and their response to LoMo epitopes, we used the RATE program (30) to calculate the relative frequency and significance of association between all the epitopes/regions and HLA alleles (or combinations thereof) expressed in responding donors. This analysis allowed inferring potential restrictions for the four of the main epitopes. It should be emphasized that these inferred alleles are meant to restrict the potential choices for the most likely restricting elements, and that further experimentation is required to conclusively assign restriction.

Table S7 in Supplementary Material lists for each of the peptides the inferred restrictions and details the number and combinations of donors that responded (R+) or did not respond (R−) to a given peptide, and the number of donors expressing (A+) or not expressing (A−) a given HLA(s). Accordingly, for example, the SSLKHPSNIAVDPIERL epitope, 100% (4/4) of the responders express the HLA DQA1*03:01, allele while none of the non-responders expressed the same HLAs (*p* = 0.008).

### Detection of Human LoMo-Specific CD4^+^ T Cells *Ex Vivo* Allows to Further Characterize Specificity and Phenotype of Responses

**Figure 5:**
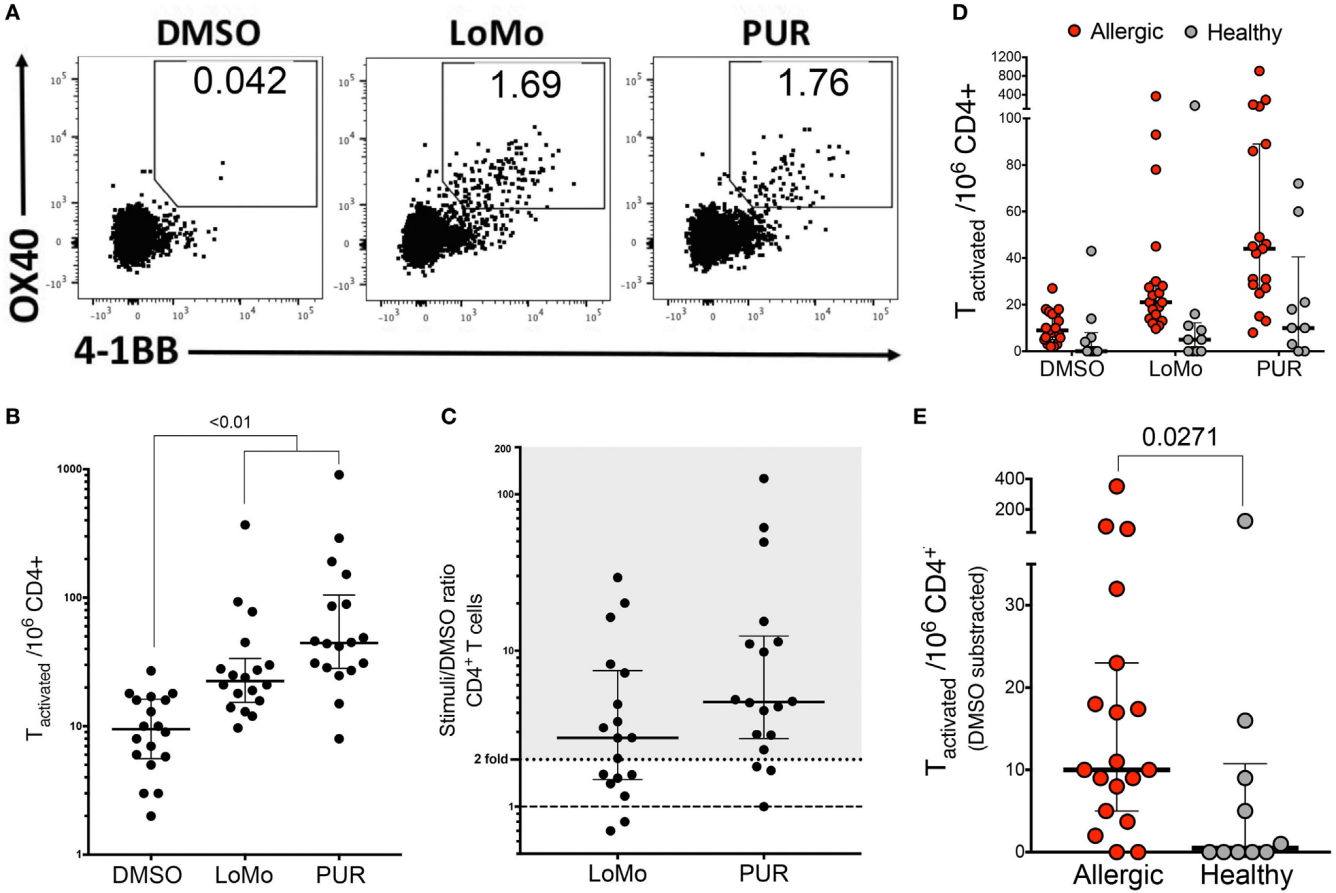
*Ex vivo* detection of human LoMo-specific CD4^+^ T cells. **(A)** Representative flow cytometry plots of 4-1BB^+^OX40^+^ upregulation by CD4^+^ T cells after stimulation with DMSO, LoMo, or PUR extract for 24 h. **(B)** Graph shows total number of stimulated 4-1BB^+^OX40^+^CD4^+^ T cells per 1 × 10^6^ PBMCs after AIM/4-1BB enrichment assays. Median ± interquartile range is represented for each stimulus. Statistical analysis was performed by Friedman’s non-parametric repeated measures comparisons. **(C)** Median of total number of activated T cells represented as fold over background (DMSO). Positive reactivity was defined as stimuli index >2-fold. Each dot represents one donor. **(D)** Total number of allergic vs. non-allergic activated CD4^+^ T cells after AIM/4-1BB enrichment assays. Median ± interquartile range is represented for each stimuli **(E)** Median of total number of LoMo-specific T cells subtracted over background (DMSO). Each dot represents one donor. Statistical analysis was performed by two-tailed Mann–Whitney test.

To further establish the biological relevance of these observations and to exclude the possibility that the responses detected were an artifact induced by *in vitro* expansion, we tested a megapool consisting of all 225 LoMo peptides for its capacity to induce *ex vivo* T cell activation. For this purpose, cells were stimulated for 24 h with LoMo megapool followed by enrichment for the activation marker 4-1BB. Subsequently, enriched cells were stained with an antibody cocktail against activation and memory-specific surface markers, and T cell activation was determined by flow cytometry, assessing dual expression of 4-1BB and OX40 (Figure 5A).

Here, T cell responses were measured directly *ex viv*o, and therefore the data allow to compare the relative magnitude and frequency of responses of T cells specific for either LoMo peptides or the high molecular extract (PUR) fraction of the urine. LoMo responses were detected in 11/18 donors. To further investigate the contribution of LoMo-specific T cell responses to mouse allergy, we also tested *ex vivo* T cell activation to the high molecular weight (>3 kDa) mouse urine extract (PUR). The PUR extract elicited the highest reactivity in 15/18 donors (83%). By comparison, responses against the LoMo pool were seen in 61% of the donors (Figure 5). In terms of magnitude, PUR extract reactivity was associated with a higher reactivity of responses, with a median SI of 4.7 (Figure 5C). The responses against the LoMo pool had a median SI of 2.8 (Figure 5C). In conclusion, LoMo reactivity was, albeit lower than PUR reactivity, fairly comparable in frequency and magnitude.

The *ex vivo* assay modality was further used to address the specificity of responses, namely whether LoMo responses were specifically associated with donors sensitized to mouse allergens. Accordingly, we also tested a group of subjects non-allergic to mouse allergens. As expected few responses and little reactivity were detected (Figure 5D), with a response rate of 2/10 donors and a median SI of 1.02. As shown in Figure 5E, the number of LoMo reactive CD4^+^ T cells is significantly lower than observed in the mouse allergic counterparts (*p* = 0.0271).

**Figure 6:**
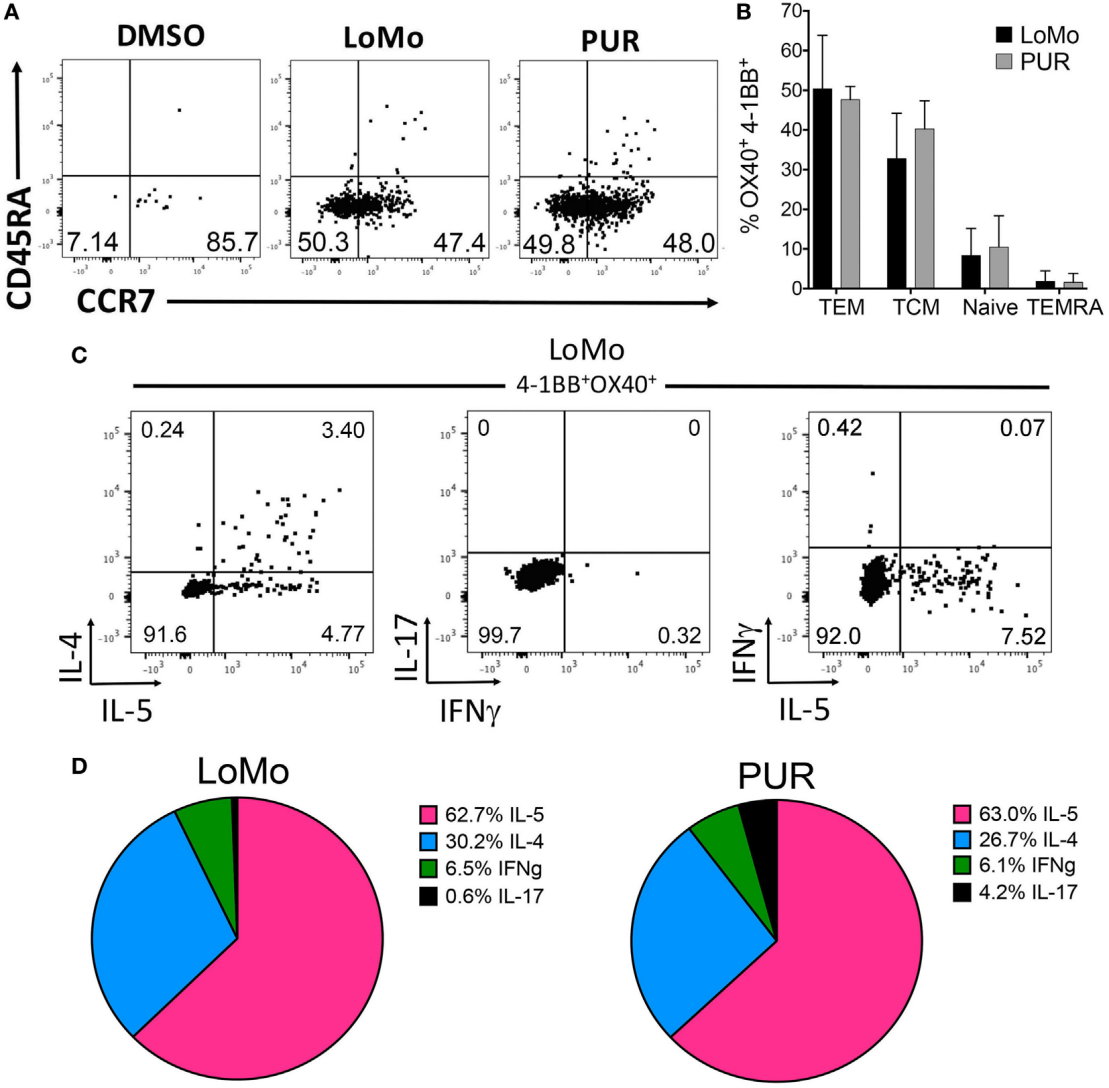
Phenotype and polarization of human LoMo-specific CD4^+^ T cells *ex vivo*. **(A)** Representative flow cytometry plots of 4-1BB^+^OX40^+^CD4^+^ T cells gated on CD45RA and CCR7 expression after 24 h of stimulation. **(B)** Graph shows frequency of central memory (CD45RA^−^CCR7^+^), effector memory (CD45RA^−^CCR7^−^), naïve (CD45RA^+^CCR7^+^), and TEMRA (CD45RA^+^CCR7^−^) cells. Median ± interquartile range is represented for each population. **(C)** Representative flow cytometry plots of 4-1BB^+^OX40^+^CD4^+^ T cells LoMo-specific cytokine production after AIM and ICS assays combined. **(D)** Patterns of cytokine production in LoMo or PUR antigen-specific T cells expressed as frequency of each individual cytokine (*n* = 4) donors.

Finally, LoMo-specific CD4^+^ T cell reactivity was associated with a memory phenotype involving both central (TCM, CD45RA^−^CCR7^+^) and effector (TEM, CD45RA^−^CCR7^−^) memory subsets (Figure 6A). The relative frequency of TCM or TEM was not significantly different between LoMo and PUR stimulation (Figure 6B). As expected, and paralleling *in vitro* restimulation results, IL-5 along with IL-4 was the cytokine most abundantly secreted after LoMo stimulation, as detected by intracellular staining (Figure 6C). No significant differences were detected in the polarization of responses between LoMo and PUR conditions for a limited number of donors analyzed (Figure 6D). The sum of the percentage of positive responses for IFNγ^+^IL-5^+^IL4^+^IL-17 for each donor was used to define the 100% of responses value. The relative contribution of each cytokine is represented in the pie chart as the fraction of responses accounted by each cytokine. Admittedly, double-positive responses cannot be extrapolated from this representation. In general, as shown for a representative donor, cells producing both IL-4 and IL-5 were common (Figure 6C, left), while no overlap existed between cells producing IFNγ and IL-17 (Figure 6C, middle), or IFNγ and IL-5 (Figure 6C, right). These results indicate that the LoMo-specific CD4^+^ T cell reactivity is associated with Th2 memory cells.

## Discussion

Here, we report the first description of T cell recognition, in mouse allergic donors, of peptides found naturally in the low molecular weight fraction of mouse urine. This study was conceived based on the large amount of proteinaceous material found within the filtrate of purified mouse extract.

The recognition of small molecular weight material in urine by T cells from allergic donors is not completely unprecedented, as low molecular weight haptens and drug molecules have long been recognized as potent allergens, and T cell based models of allergic reactions commonly utilize these types of reagents (38–40). Future experiments addressing whether the associated T cells express skin or lung homing markers might help to determine the route of exposure of these components to the immune system.

Consistent with previous reports (9, 25), the urine peptidome detected by mass spectrometry was highly diverse, with over 1,300 distinct peptiforms in the low molecular weight fraction. Strikingly, the universe of peptides recognized by T cells was also remarkably heterogeneous, with about half of the 225 peptides tested being recognized by at least one of the allergic donors tested. This finding further underlines how the low molecular weight fraction represents a previously under-appreciated but rich source of antigenic peptides.

Posttranslationally modified peptides were abundant in the low molecular weight fraction. The most common posttranslational modification was hydroxyproline, whose predominant source was collagen. Indeed, this was already observed previously and represents a major route for collagen breakdown and excretion (33). Of relevance, hydroxyproline-containing peptides are relatively resistant to exoprotease degradation (41), which is a factor that could contribute to their immunogenicity. Immunogenic hydroxyproline peptides have previously been described in models of collagen induced arthritis (42). Here, the highest response magnitude was elicited by a hydroxyproline peptide, which was recognized in 4 out of 19 donors. Peptides derived from collagen and its precursors accounted for 9.2% of the total response.

Notably, peptides derived from proteins not classically associated with urine accounted for the highest magnitude of responses. Besides the already mentioned collagen, main contributors included Egf, Beta-globin, and Meprin A (15.9, 8.1, and 4.8% of the total response, respectively). Interestingly, although no Egf peptides were recognized by donors expanded with PUR or EPTH extract, Egf-derived peptides were recognized by more than half (10/19) of the LoMo-expanded donors. This further highlights the low molecular weight fraction’s potential to be a unique source of antigenic peptides that may otherwise be missed when using traditional extracts. These results also highlight that the systemic degradation of a diverse set of proteins might contribute peptides that escape renal reabsorption and contribute to the immune-urine peptidome.

Conversely, and as expected, among the peptides with highest response frequency were the peptides derived from proteins classically associated with mouse urine (MUPs, KAP, and uromodulin). This raises the possibility that the sensitization of T cells through urine peptides might contribute to the overall reactivity to urinary mouse proteins.

Analysis of the polarization of cytokine production revealed that responses were dominated by IL-5 (63.1%), suggesting that the detected T cell reactivity conforms to the classical functional phenotype of allergy-associated T cells.

Following a previously described approach (43), we created a single pool of urine oligopeptides, termed low molecular weight megapool (LoMo), consisting of the 225 peptides and assessed its ability to elicit T cell reactivity from mouse allergic donors directly *ex vivo* (31). Specifically, following enrichment based on 4-1BB expression, we assayed for upregulation of the activation markers OX40 and 4-1BB as a read-out for peptide-specific T cell reactivity (44). This approach demonstrates that LoMo-specific T cells can be detected without any *in vitro* expansion. Importantly, LoMo-specific T cell responses were significantly lower or absent in a control cohort of non-allergic donors. These observations formally establish the specificity of the responses, by being associated with allergic donors, and absent or marginal in non-allergic donors.

Moreover, by utilizing classical markers of memory differentiation, we could further demonstrate that LoMo-specific T cells were derived from memory compartments, implying that they were previously exposed to antigen *in vivo*. Further intracellular cytokine staining demonstrated that IL-5, similar to *in vitro* assessments, and additionally IL-4 was the dominant cytokine secreted after LoMo stimulation, which confirmed the pattern of Th2 dominance.

Finally, the results presented herein demonstrate that significant T cell reactivity exists against a set of urine oligopeptides which are expected, due to their small size, to be associated with little or no IgE reactivity. Overall, these data suggest that T cell responses against urine-derived low molecular weight peptides are a specific and previously unrecognized target of T cells from mouse allergic donors. This reactivity could have significance in terms of both understanding allergic immunopathology and potential practical applications. Furthermore, this observation could potentially be applicable to other allergen systems, since lower molecular weight material is not usually considered and analyzed for reactivity.

## Ethics Statement

Patients involved in this study were recruited from San Diego, CA, USA, and New York City, NY, USA following Institutional Review Board approval (IRB protocols: VD-112-0217, GCO 13-0691). All patients enrolled in this study provided written consent.

## Author Contributions

RA, VS, BP, and AS participated in the design and planning of the study. RA, JP, and VS performed and analyzed experiments. CM and WH performed mass spectrometry analysis. JS conducted bioinformatics analyses. PB handled clinical recruitment. RA, JP, VS, and AS wrote the article. EP and SM conducted HLA typing. All the authors have read, edited, and approved the manuscript.

## Conflict of Interest Statement

The authors declare that the research was conducted in the absence of any commercial or financial relationships that could be construed as a potential conflict of interest.
