# Supplementary figures and images for: Urinary Peptides As a Novel Source of T Cell Allergen Epitopes
Source: Front Immunol. 2018 Apr 26;9:886. doi: 10.3389/fimmu.2018.00886 (PMC5932195; doi:10.3389/fimmu.2018.00886)

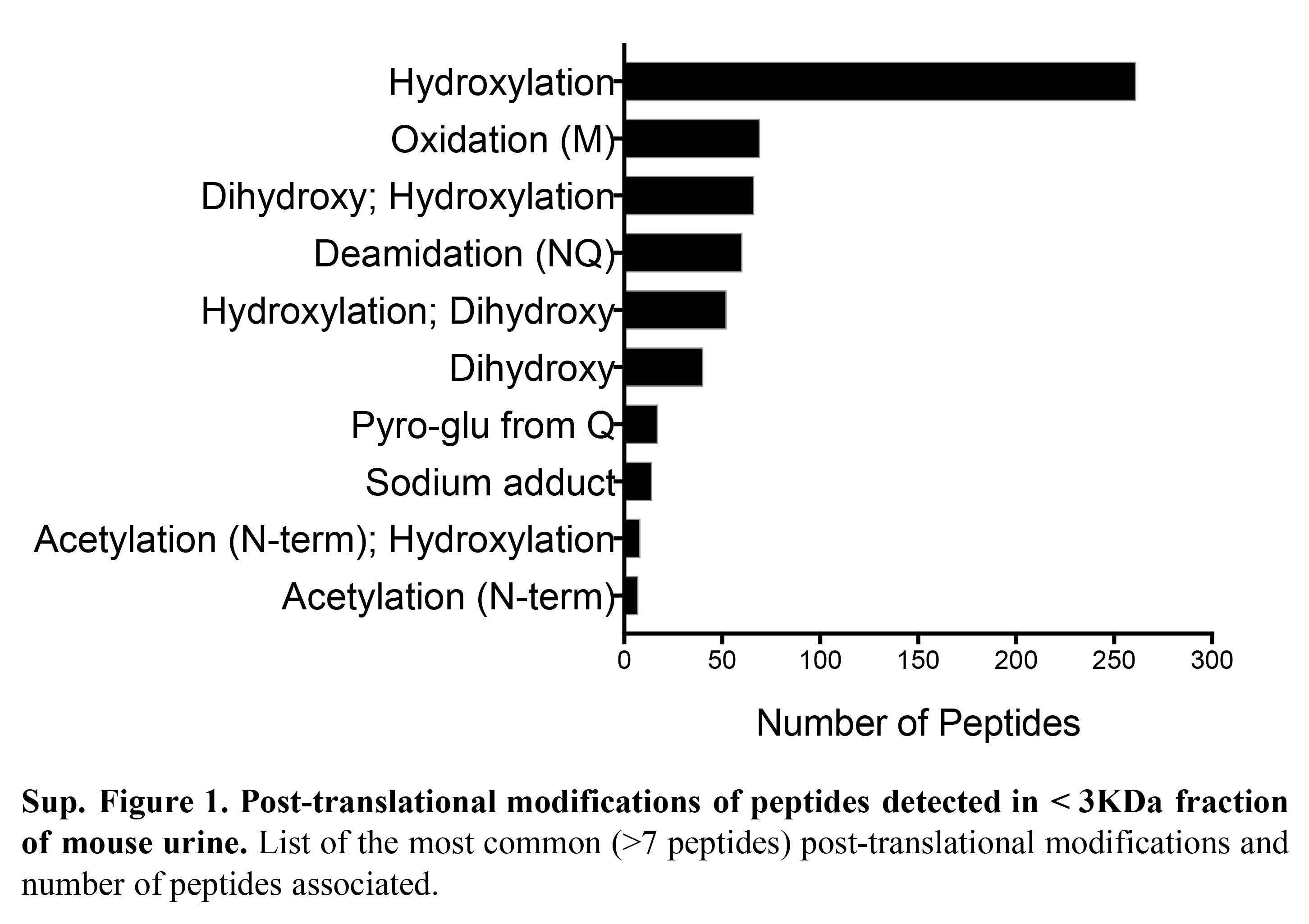

Supplement: Supplementary file 1 [file image_1.JPEG]
